# Predictors of Persistent Anaemia in the First Year of Antiretroviral Therapy: A Retrospective Cohort Study from Goma, the Democratic Republic of Congo

**DOI:** 10.1371/journal.pone.0140240

**Published:** 2015-10-16

**Authors:** Pierre Zalagile Akilimali, Espérance Kashala-Abotnes, Patou Masika Musumari, Patrick Kalambayi Kayembe, Thorkild Tylleskar, Mala Ali Mapatano

**Affiliations:** 1 Kinshasa University School of Public Health, University of Kinshasa, Kinshasa, Democratic Republic of Congo; 2 Centre for International Health, University of Bergen, Bergen, Norway; 3 Department of Global Health and Socio-Epidemiology, Kyoto University School of Public Health, Kyoto, Japan; CEA, FRANCE

## Abstract

**Background:**

Anaemia is associated with adverse outcomes including early death in the first year of antiretroviral therapy (ART). This study reports on the factors associated with persistent anaemia among HIV-infected patients initiating ART in the Democratic Republic of Congo (DR Congo).

**Methods:**

We conducted a retrospective cohort study and analyzed data from patients receiving HIV care between January 2004 and December 2012 at two major hospitals in Goma, DR Congo. Haemoglobin concentrations of all patients on ART regimen were obtained prior to and within one year of ART initiation. A logistic regression model was used to identify the predictors of persistent anaemia after 12 months of ART.

**Results:**

Of 756 patients, 69% of patients were anaemic (IC95%: 65.7–72.3) at baseline. After 12 months of follow up, there was a 1.2 g/dl average increase of haemoglobin concentration (P < 0.001) with differences depending on the therapeutic regimen. Patients who received zidovudine (AZT) gained less than those who did not receive AZT (0.99 g/dl vs 1.33 g/dl; p< 0.001). Among 445 patient who had anaemia at the beginning, 33% (147/445) had the condition resolved. Among patients with anaemia at ART initiation, those who did not receive cotrimoxazole prophylaxis before starting ART(AOR 3.89; 95% CI 2.09–7.25; P < 0.001) and a AZT initial regimen (AOR 2.19; 95% CI 1.36–3.52; P < 0.001) were significantly at risk of persistent anaemia.

**Conclusions:**

More than two thirds of patients had anaemia at baseline. The AZT-containing regimen and absence of cotrimoxazole prophylaxis before starting ART were associated with persistent anaemia 12 months, after initiation of treatment. Considering the large proportion of patients with persistence of anaemia at 12 months, we suggest that it is necessary to conduct a large study to assess anaemia among HIV-infected patients in Goma.

## Background

Anaemia during infection with the human immunodeficiency virus (HIV) may have multiple causes [1]. The prevalence of anaemia in people with acquired immunodeficiency syndrome (AIDS) has been estimated between 63 to 95% [2–3].

The incidence of anaemia increases with the progression of HIV infection [4–6]. Anaemia is also a known feature of certain opportunistic infections, includingas tuberculosis, atypical mycobacteria, microcystosis, cryptococcus and parvovirus B19 [7]. It has been suggested that the use of a regimen containing zidovudine (AZT) at the initiation of antiretroviral therapy (ART) is associated with the incidence of anaemia, with bone marrow toxicity being postulated as the main underlying mechanism [8]

In addition to reduced physical functioning and quality of life caused by anaemia, its presence at the initiation of ART has been associated with HIV disease progression and mortality [4, 9–14]. Indeed, in the Euro SIDA cohort, patients with severe anaemia at baseline had 13 times greater risk of death during the first year of ART than patients with a normal haemoglobin (Hb) concentration [10], similar findings having been reported from Tanzania, Côte d'Ivoire, South Africa, Malawi and the Democratic Republic of Congo (DR Congo) [9, 13–15].

There has been a decline in the prevalence of anaemia and an increment in mean CD4+ T cell count among HIV infected patients after ART initiation, as seen from studies carried out in Africa, such as Adane’s study [16]. Research in Europe and North America has also shown that ART itself can be an effective treatment of anaemia associated with HIV infection [7, 10, 17], just as improvement of haemoglobin concentration occurs with ART [9, 14]. However, only a few studies have looked at the changes of haemoglobin concentration among patients on ART in resource-limited settings, and whether these changes may vary with the ART regimen. Given the number of patients on ART in this rural area selected for study in the DR Congo, we believe that a better understanding of the role of anaemia in HIV treatment is critical to developing strategies to reduce morbidity and improve survival on ART.

Mortality is higher in the first year of ART, with anaemia being cited as one of the factors of death among patients receiving ART [10, 15]. The present study has been aimed at determining the predictors of persistence of anaemia during the first year of treatment with ART among HIV patients in two hospitals in Goma (DR Congo).

## Methods

### Study site and design

We conducted a retrospective cohort study on patients from two major hospitals, the Virunga hospital and Goma provincial referral hospital (GPRH) in Goma. This is a city located in the eastern part of DR Congo that has been affected by civil war for many years, and which has a prevalence of HIV of 0.9% [18]. HIV care and treatment services at the Virunga hospital and GPRH have provided ART free of charge since 2004.

### Study population

We describe the baseline haematological profile in a cohort of treatment-naïve individuals aged 15 years or older. These patients were enrolled in the HIV care and treatment programme at the Virunga hospital and GPRH between January 2004 and December 2012.

#### Treatment, monitoring and information collected

In DR Congo, including Goma, ART was initiated in accordance with guidelines from the World Health Organization (WHO) and the National AIDS Control Program [19–20]. First-line treatment comprised Stavudine (D4T) or AZT, combined with Lamivudine (3TC), and either Nevirapine (NVP) or Efavirenz (EFV). Regimen choice was subject to availability, with use of a generic fixed-dose combination of D4T, 3TC and NVP whenever possible. Patients were seen by a clinical officer every 3 months. Their CD4 cell and full blood (including haemoglobin) counts were scheduled every 3 months as part of a routine follow- up.

Anaemia was defined according to the WHO guidelines, as a haemoglobin concentration of <12 g/dl for women and <13 g/dl for men [21]. We classified the anaemia as mild (11–11.9 g/dl for women and 11–12.9 g/dl for men), moderate (8–10.9 g/dl) and severe (<8 g/dl in both genders).

The following informations were collected: socio-demographic characteristics (age, gender, marital status and education), clinical characteristics (therapeutic regimen, WHO clinical stage of disease) and biological characteristics (CD4 count, haemoglobin at baseline and 12 months after treatment initiation). The outcome, one year after the start of ART was the persistence of anaemia. Loss to follow-up was defined as failure to return to the centre 3 months after the last clinic visit. Mortality was ascertained through family or hospital report as well as, active tracing.

All patients who were anaemic at the time of ART initiation and had a follow-up haemoglobin measurement 12 months later were included in the analysis.

### Statistical analysis

In general, we applied the value closest to starting ART as the baseline value. Chi-square tests were used to study the association of baseline characteristics with severity of anaemia at baseline. The mean of the haemoglobin concentration and its confidence interval were calculated. Paired samples t-tests were used to compare haemoglobin concentration before and 12 months after ART initiation. Overall changes in haemoglobin concentration on treatment were further examined by stratifying results by sex (male versus female) at treatment initiation using the t-test. The paired t-test was used to compare the haemoglobin gain over 12 months between the group receiving AZT and those not receiving AZT. McNemar’s test was used to compare the proportion of patients with anaemia at the beginning and end of the first 12 months on ART.

A logistic regression model was used to identify predictors of persistent anaemia after 12 months of ART among anaemic patients at treatment initiation. Gender, age, stage of disease, ART regimen, cotrimoxazole prophylaxis and CD4 count at baseline were entered into a logistic model to find the factors associated with the persistence of anaemia. Data were analysed with SPSS version 21.0 for Windows. All tests were two-sided and the level of significance was set at P < 0.05.

### Ethical Statement

This study was approved by the institutional review board ethics committee for research subjects at Kinshasa University School of Public Health. Written informed consent was not given by participants for their clinical records to be used in this study, but patient records/information was anonymized and de-identified prior to analysis. (No App: ESP/CE/034/14 of 27.08.2014).

## Results

### Patient characteristics at enrollment

**Fig 1:**
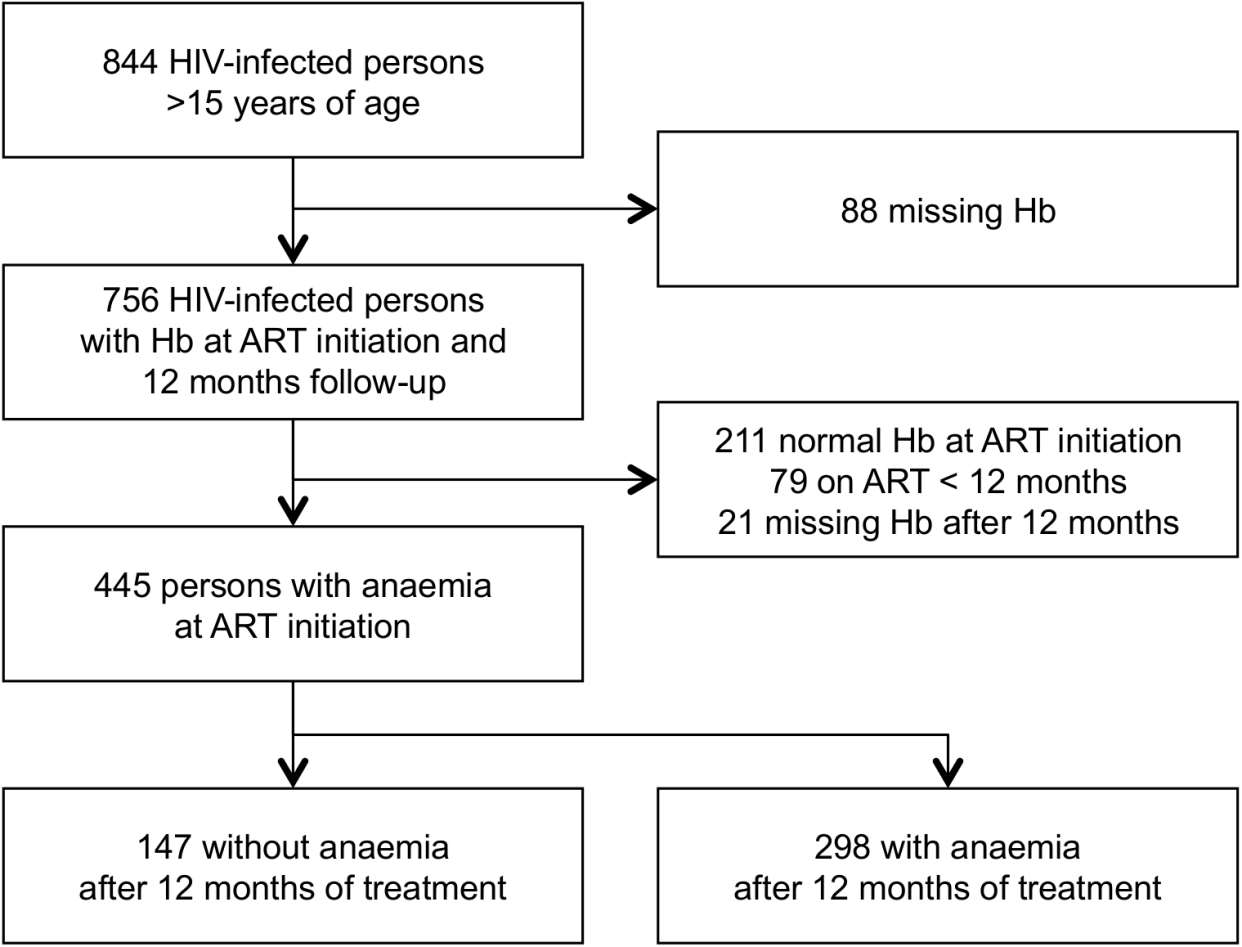
Study flow of participants in two different HIV treatment centers at Goma, in the Democratic Republic of Congo.

A total of 844 HIV patients were enrolled in the HIV program between January 1, 2004, and December 15, 2012. We excluded those who had not yet been on ART for 12 months (79 patients) and those with missing Hb values at enrolment (88 patients). Overall, the excluded patients were similar to those who were kept in the analysis in terms of age (mean 39.51± 9.79 years) and gender (63.9% female) distributions. Among 445 patients who had anaemia at the beginning, 33% (147/445) had resolution of anaemia (See Fig 1).

A total of 756 HIV-infected subjects who were eligible for this study and included in this study (Fig 1). The mean age was 38.76 years (standard deviation [SD] 9.96) and 493 patients (66.5%) were women. At baseline, 109 patients (14.6%) had clinical AIDS (WHO stage 4), 418 (56.0%) had WHO stage 3, 164 (22%) had WHO stage 2, and 54 (7.4%) had WHO stage 1 disease. Sixty nine percent of patients were anaemic (95% CI: 65.7–72.3), of which 45.1, 17.7, and 6.5% had mild, moderate, and severe anaemia, respectively.

Table 1 gives an overview of patient characteristics at enrolment and associations with anaemia. Male gender, clinical AIDS (stage of disease) and low CD4 count were all significantly associated with anaemia at enrolment.

**Table 1:** Patient characteristics and degree of anaemia at enrolment.

	Overall (n)	No anaemia	Mild anaemia	Moderate anaemia	severe anaemia
**All**	756(100)	228(30.2)	201(26.6)	278(36.8)	49(6.5)
**Age (years)** *					
≤ 38	372(49.2)	109(47.8)	92(45.8)	154(55.4)	17(34.7)
> 38	384(50.8)	119(52.2)	109(54.2)	124(44.6)	32(65.3)
**Gender**					
male	248(32.8)	64(28.1)	95(47.3)	65(23.4)	24(49.0)
female	493(65.2)	162(71.1)	97(48.3)	209(75.2)	25(51.0)
missing	15(2.0)	2(0.9)	9(4.5)	14(1.4)	0(0.0)
**Education level atteined**					
None	287(38.0)	87(38.2)	70(34.8)	110(39.6)	20(40.8)
primary	249(32.9)	81(35.5)	72(35.8)	86(30.9)	10(20.4)
Secondary or higher	197(26.1)	55(24.1)	53(26.4)	72(25.9)	17(34.7)
missing	23(3.0)	5(2.2)	6(3.0)	10(3.6)	2(4.1)
**Marital status**					
married/Cohabitating	384(50.8)	128(56.1)	112(55.7)	120(43.2)	24(49.0)
Single/Divorced/separated/widow(ed)	366(48.4)	99(43.4)	87(43.3)	156(56.1)	24(49.0)
missing	6(0.8)	1(0.4)	2(1.0)	2(0.7)	1(2.0)
**stage of desease (WHO)**					
StageI or II	219(29.0)	75(32.9)	54(26.9)	77(27.7)	13(26.5)
Stage III	418(55.3)	131(57.5)	119(59.2)	143(51.4)	25(51.0)
Stage IV	109(14.4)	19(8.3)	25(12.4)	54(19.4)	11(22.4)
missing	10(1.3)	3(1.3)	3(1.5)	4(1.4)	0(0.0)
**CD4 (cells/μl)**					
< 50	154(20.4)	39(17.1)	38(18.9)	61(21.9)	16(32.7)
50 to 199	337(44.6)	97(42.5)	93(46.3)	124(44.6)	23(46.9)
≥ 200	242(32.0)	86(37.7)	61(30.3)	88(31.7)	7(14.3)
missing	23(3.0)	6(2.6)	9(4.5)	5(1.8)	3(6.1)
**Cotrimoxazole**					
no	618(81.7)	192(84.2)	159(79.1)	226(81.3)	41(83.7)
yes	109(14.4)	30(13.2)	33(16.4)	41(14.7)	5(10.2)
missing	29(3.9)	6(2.6)	9(4.5)	11(4.0)	3(6.1)
**Haemoglobin, mean ± SD**	10,9 **±** 2,0	13.2 **±** 1.2	11.5 **±** 0.5	9.6 **±** 0.8	6.9 **±** 0.9

SD: standard deviation

*: median age was 38 years

### Haemoglobin evolution during antiretroviral treatment

Of the 656 patients with documented haemoglobin concentrations at initiation of ART and the end of the first 12 months, 67.3% (445/656) were anaemic at initiation. Twelve months later, the proportion of patients with anaemia was reduced to 43% (308/656 McNemar’s test, p < 0.001) (See S1 Table, which shows evolution of proportion of anaemia at the time of initiation of ART and 12 months later).

At ART initiation the mean haemoglobin was 11.06 g/dl (SD 2.06), with women having a lower haemoglobin than men (10.83 vs 11.28; p = 0.010). After receiving ART for 12 months, the mean haemoglobin increased to 12.31 g/dl (SD 1.96) (See S2 Table, which shows haemoglobin evolution during antiretroviral treatment), an average increase of 1.2 g/dl (p < 0.001). The average differed depending on the therapeutic regimen: patients who received AZT gained less than those who did not receive AZT (0.99 g/dl vs. 1.33 g/dl; p< 0.001) (See S2 Table).

### Predictors of persistent anaemia among 445 HIV-infected adults anaemic at ART initiation

Two hundred thirty-two patients who did not have anaemia at the time of ART initiation, and 79 patients who were anaemic at enrolment and received ART for less than 12 months were excluded in the logistic regression model. Among patients who were anaemic at ART initiation, the absence of cotrimoxazole prophylaxis prior to initiating ART [adjusted odds ratio (AOR) 3.89; 95% CI 2.09–7.25; P < 0.001] and an AZT-containing initial regimen (AOR 2.19; 95% CI 1.36–3.52; P < 0.001) were significantly associated with the persistence of anaemia (Tables 2 and 3). The database for this manuscript is available as an additional supporting information (S1 and S2 Datasets).

**Table 2:** Bivariate analysis of factors associated with persistent anaemia.

	Persistent anaemia		Crude OR (95%CI)	p-value
	yes	no		
**Age (years)** *				
- ≤ 38	153(67.4)	74(32.6)	1	
- > 38	145(66.5)	73(33.5)	0.96 (0.65–1.43)	0,842
**Gender**				
- female	196(65.8)	101(34.2)	1	
- male	96(69.6)	42(30.4)	1.19 (0.77–1.84)	0,434
**WHO stage**				
- Stage I or II	83(65.4)	44(34.6)	1	
- Stage III	155(64.9)	84(35.1)	0.98 (0.62–1.54)	0,924
- Stage IV	55(75.3)	18(24.7)	1.62 (0.85–3.09)	0,143
**CD4 (cells/μl)**				
- ≥ 200	95(69.3)	42(30.7)	1	
- 50 to 199	136(66.3)	69(33.7)	0.87 (0.55–1.39)	0,561
- < 50	62(68.9)	28(31.1)	0.98 (0.55–1.74)	0,942
**Cotrimoxazole**				
- yes	25(43.1)	33(56.9)	1	
- no	259(70.6)	108(29.4)	3.17 (1.80–5.58)	< 0,001
**Regimen**				
- Without AZT	161(60.3)	106(39.7)	1	
- With AZT	134(77.9)	38(22.1)	2.32 (1.50–3.59)	< 0,001

*: Median age was 38 years

**Table 3:** Multivariate analysis of factors associated with persistent anaemia.

	Crude OR (95%CI)	p	Adjusted OR (95%CI)	p
**Age (year)**				
- > 38 (vs ≤ 38)	0.96 (0.65–1.43)	0.842	0.94 (0.60–1.47)	0.793
**Gender**				
- male(vs female)	1.19 (0.77–1.84)	0.434	1.14 (0.69–1.85)	0.609
**WHO stage**				
- Stage III(vs stage I or II)	0.98 (0.62–1.54)	0.924	0.83 (0.49–1.39)	0.474
- Stage IV(vs stage I or II)	1.62 (0.85–3.09)	0.143	1.59 (0.76–3.33)	0.215
**CD4(cells/μl)**				
- 50 to 199(vs ≥ 200)	0.87 (0.55–1.39)	0.561	1.21 (0.72–2.02)	0.468
- < 50(vs ≥ 200)	0.98 (0.55–1.74)	0.942	1.22 (0.64–2.33)	0.555
**Cotrimoxazole**				
- no (vs yes)	3.17 (1.80–5.58)	< 0.001	3.89 (2.09–7.25)	<0.001
**Regimen**				
- with AZT(vs without AZT)	2.32 (1.50–3.59)	< 0.001	2.19 (1.36–3.52)	0.001

We have introduced in the model Age, Gender, WHO stage, CD4, Cotrimoxazole and regimen.

## Discussion

In this study 69% of HIV-infected subjects had anaemia at the time they had been enrolled in HIV care. Women were more likely than men to be anaemic. Patients who were not on cotrimoxazole prophylaxis before ART initiation and those on an AZT-containing regimen were more likely to have persistent anaemia 12 months after initiation of ART. Among 445 patients with anaemia from the start, 33% had resolution of anaemia.

Haemoglobin concentration increased significantly in patients who received ART; on average the haemoglobin increased 1.2 g/dl over the first 12 months. European and North American studies reported that ART is associated with resolution of HIV-associated anaemia [10, 17]. More recently, similar findings come from work in sub-Saharan Africa; in rural Uganda found it was shown that the mean haemoglobin increased from 11.3 g/dl at baseline to 12.8 g/dl after 12 months on ART [22]. However, the magnitude of the increased haemoglobin might vary depending on its baseline concentration and the degree of immunodeficiency. Our study is consistent with others showing that HIV-associated anaemia can be reversed with the use of ART as in the research carried out by Moore et al. (17). To a certain degree indicates that HIV-associated anaemia in our setting is mainly the result of factors related to the HIV infection itself, such as chronic inflammation and opportunistic infections, rather than tropical diseases or specific environmental factors. Sixty-seven percent of patients having persistent anaemia at 12 months represents a large proportion, the possible causes being poor nutrition, drug toxicity and parasitic diseases. But we did not measure such data, which is one of the limitations of the study.

Although we observed an increase in haemoglobin over the 12 months follow up, differentials exist depending on the ART regimen, patients on AZT-based regimen experiencing a lower increase in haemoglobin compared to those on D4T-based regimen. Similar findings were reported from Uganda where anaemic patients on D4T- containing regimens had a significantly larger increase in haemoglobin than those on AZT-based regimens [23]. The same pattern was shown in a meta-analysis of six randomized trials, in which the mean haemoglobin concentration was 0.8 g/dl lower in patients who received AZT than those who received D4T after 48 weeks on ART [4]. However, the trend of improving haemoglobin concentration despite initiating an AZT-containing regimen has been similarly documented in other studies from Ugandan [23] and South African cohorts [8, 24].

We also found that persistent anaemia was associated with ART regimen and chemoprophylaxis of cotrimoxazole. Patients who had an initial AZT-containing regimen were more likely to have persistent anaemia, which is in line with a previous cohort study in a rural hospital in Tanzania showing that the majority of patients who were anaemic at the time of ART initiation had significantly increased haemoglobin levels over the initial 12 months of ART. However, patients on AZT-based regimens had more risk of persistent anaemia [25]. There is a widely established link between the use of AZT and anaemia through myelo suppression [26, 27]. AZT can inhibit bone marrow activity, which leads to decreased production of blood cells and platelets [28, 29]. Although, the association between AZT use and anaemia is well documented [25–31], its clinical significance remains under debate. The threshold defining anaemia and study designs have differed in research methods of assessing the link between anaemia and ART regimens. Indeed the Human Immunodeficiency Virus Epidemiology Research Study [32] showed that, although the use of AZT was associated with an increased risk of anaemia (defined as a haemoglobin concentration of <12 g/dl) in the pre-HAART era (1993–1996), use of AZT during the HAART era (1996–2000) was not significantly associated with anaemia. In contrast, the Women’s Interagency HIV Study recorded the presence of anaemia (defined as a haemoglobin concentration of <10 g/dl or a physician's diagnosis) in 41.6% of subjects receiving AZT compared with 34.3% in those not receiving AZT [33].

Our results also support findings that ART improves haemoglobin regardless of the regimen type (AZT containing versus non-AZT containing) and the degree of immuno-suppression; however, correction of anaemia does not have the same magnitude depending on the therapeutic regimen received by the patient (AZT-based vs non- AZT-based). These results underscore the possible caveats of AZT, and suggest that other drugs might be preferable in anaemic patients, especially in countries such as DR Congo where anaemia is endemic, and probably related to malaria, intestinal parasites, malnutrition and other causes. The 2013 revision of the WHO guidelines recommended tenofovir (TDF) in combination with 3TC or FTC and a non-nucleoside reverse transcriptase inhibitor as the first regimen option in individuals initiating ART unless contraindicated [34].

An unexpected finding was the negative association between the prescription of cotrimoxazole and anaemia. Its use is known to be associated with aplastic anaemia or immune-mediated destruction of specific populations of blood cells [35]; but our patients who were on cotrimoxazole prior to initiating ART were less likely to have anaemia at baseline, and less likely to be anaemic after 12 months. We assume that the protective effect of cotrimoxazole in preventing opportunistic conditions such as the Mycobacterium avium complex [36], bacterial septicemia, or other conditions well known to cause anaemia as a result of the chronic inflammation process, could have outweighed the risk of cotrimoxazole in inducing anaemia. Management of anaemia in settings with a high prevalence of HIV should always include an HIV test, and ART should be initiated taking into account other local causes of anaemia. Our study suggests that this would be sufficient to correct the anaemia in most subjects, and that additional test to identify patients in whom additional treatment (such as iron supplementation or oxidative stress correction) might be required.

Regarding several limitations in the present study, the first is related to the technique used to collect data. Indeed, with the review of patient records, some information may have been recorded with little rigor or accuracy. Second, we did not take into account other factors such as adherence to treatment, transfusion history and the presence of co-morbidities (nutritional status, HIV-tuberculosis co-infection, intestinal parasites, microcystosis, malaria etc.). Nevertheless, this study has the advantage of being among the few to document factors associated with persistent anaemia during the first year of ART not only in the DR Congo but in an area with ongoing conflict for several years.

## Conclusion

Over two-thirds of patients had anaemia at baseline. An AZT-containing regimen and absence of cotrimoxazole prophylaxis before starting ART were associated with persistent anaemia 12 months after initiation of treatment. Considering the large proportion of patients with persistence of anaemia at 12 months, we suggest that it is necessary to conduct a large study to assess the anaemia among HIV-infected patients in Goma.

## Supporting Information

S1 Dataset The Excel database used for this manuscript. (XLS)

S2 Dataset The Stata database used for this manuscript. (DTA)

S1 Table Evolution of proportion of Anaemia at the time of initiation of ART and 12 months later. (DOCX)

S2 Table Haemoglobin evolution during antiretroviral treatment (Mean with 95% Confidence Interval (g/dl)). (DOCX)
