# Supplementary material for: Predictors of Persistent Anaemia in the First Year of Antiretroviral Therapy: A Retrospective Cohort Study from Goma, the Democratic Republic of Congo
Source: PLoS One. 2015 Oct 16;10(10):e0140240. doi: 10.1371/journal.pone.0140240 (PMC4608787; doi:10.1371/journal.pone.0140240)
Supplement: S1 Table — (DOCX) [file pone.0140240.s003.docx]

S1 Table: Evolution of proportion of Anaemia at the time of initiation of ART and 12 months later

| Anaemia at the time of ART initiation | Anaemia after 12 months | | total |
| --- | --- | --- | --- |
|  | yes | no |  |
| yes | 298 | 147 | 445 |
| no | 10 | 201 | 211 |
| total | 308 | 348 | 656 |
