# Supplementary material for: Predictors of Persistent Anaemia in the First Year of Antiretroviral Therapy: A Retrospective Cohort Study from Goma, the Democratic Republic of Congo
Source: PLoS One. 2015 Oct 16;10(10):e0140240. doi: 10.1371/journal.pone.0140240 (PMC4608787; doi:10.1371/journal.pone.0140240)
Supplement: S2 Table — (DOCX) [file pone.0140240.s004.docx]

S2 Table: Haemoglobin evolution during antiretroviral treatment
(Mean with 95% Confidence Interval (g/dl))

| Variables | Hb at the beginning | p-value | Hb after 12 months | p-value | gain | p-value |
| --- | --- | --- | --- | --- | --- | --- |
| Gender* |  | 0.01 |  | <0.001 |  | 0.102 |
| - male | 11.3(11.0 -11.6) |  | 10.8(10.7-10.9) |  | 1.3 (1.1 -1.5) |  |
| - female | 10.8(10.7-10.9) |  | 12.1(11.9-12.2) |  | 1.2 (1.1 - 1.3) |  |
| Regimen* |  | 0.018 |  | <0.001 |  | <0.001 |
| - with AZT | 10.8 (10.6 -10.9) |  | 11.9 (11.7 - 12.2) |  | 0.99(0.9-1.1) | |
| - without AZT | 11.1 (11.0 -11.3) |  | 12.5 (12.3 -12.7) |  | 1.3(1.2-1.5) |  |
| Age (years)* |  | 0.271 |  | 0.139 |  | 0.454 |
| - ≤ 38 | 10.9 (10.7 - 11.1) |  | 12.2 (11.9 -12.4) |  | 1.2 (1.1-1.4) |  |
| - > 38 | 11.1 (10.9 - 11.3) |  | 12.4 (12.2 - 12.6) |  | 1.2 (1.1 -1.3) |  |
| CD4 (cells/μl)** |  | 0.001 |  | 0.077 |  | 0.653 |
| - ≥ 200 | 11.4 (11.1 -11.7) |  | 12.6 (12.3 - 12.9) |  | 1.2 (1.1 -1.3) |  |
| - 50 to 199 | 10.9 (10.7 - 11.1) |  | 12.2 (12.0 - 12.4) |  | 1.3 (1.1 -1.4) |  |
| - < 50 | 10.6 (10.3 - 10.9) |  | 12.1 (11.8 -12.4) |  | 1.2 (0.9 -1.4) |  |
| overall | 11.0(10.8-11.1) |  | 12.3(12.2-12.5) |  | 1.2(1.1-1.3) |  |

*: means were compared by using t-test; **: means were compared by using the one way Anova. Hb: Haemoglobin
